# Cataract surgery by appointment – a pilot study

**DOI:** 10.1186/1471-2415-6-18

**Published:** 2006-04-20

**Authors:** Ioannis Mavrikakis, Tassos Georgiou, Bobby Paul, Christopher SC Liu

**Affiliations:** 1 Sussex Eye Hospital, Brighton and Sussex University Hospitals NHS Trust, Brighton, UK; 2 Tongdean Eye Clinic, Brighton & Hove, UK

## Abstract

**Background:**

"Cataract Surgery by Appointment" is a new method of delivery of cataract surgery that reduces the time a patient spends in hospital by their direct arrival at the operating theatre, having self-prepared for surgery, thus avoiding admission to the ward or time spent in the Day Case Unit. The patient can stay as little as 20 minutes from their arrival to going home. We describe the process in detail, and seek to evaluate the visual outcome, safety and patient satisfaction of same.

**Methods:**

Visual outcome and safety data were obtained from patients' medical records, prospectively. Patients were also surveyed by a questionnaire to determine their satisfaction with the service and viability as a prospect for providing a more efficient cataract surgery service.

**Results:**

In 2002, fifty-one eyes of 39 consecutive patients underwent "Cataract Surgery by Appointment". There were 16 male and 23 female. The pre-operative best-corrected visual acuity was 6/9 or better in 17 (33%) eyes. The post-operative best-corrected visual acuity was 6/9 or better in 44 (86%) eyes. There were no per-operative complications. Post-operative complications occurred in 3 (6%) eyes. The average number of days from surgery to final discharge was 14.5 days.

Twenty-eight (72%) completed questionnaires were returned. The results show that the majority of patients were satisfied with their overall experience of this mode of delivery for cataract surgery.

**Conclusion:**

"Cataract Surgery by Appointment" performed under local anaesthesia by a skilled ophthalmic surgeon appears to be safe and effective for highly selected cases. This method of delivery gave a high level of patient satisfaction, and is the ultimate form of day case cataract surgery. The method may gain widespread use should per-operative intracameral pupil dilatation prove to be effective and acceptable. Attention should be paid to risk-stratification, so complex cases are allocated more time on the operating list.

## Background

Phacoemulsification with intraocular lens (IOL) implantation is the standard treatment for visually significant cataract. In the United Kingdom, cataract surgery is usually carried out in a hospital as a day case procedure, without an overnight stay. Typically, patients for uncomplicated day case cataract surgery undergo at least 4 appointments; one clinical visit, one pre-assessment visit, the operation, and one post-operative visit [1]. At the day of the operation, the patients are admitted 2–3 hours, or longer, before the time of their surgery. A nurse cares for the patient from admission to discharge. Dilating drops are instilled into the patient's eye to be operated on for at least 1 hour before the surgery. On return from theatres, the patient remains in a chair and is ready to go home within a few hours. The time from admission to discharge can be up to seven hours.

We describe a new method of delivery of cataract surgery that reduces patient waiting in the hospital by their direct arrival at the operating theatre, avoiding admission to the ward or time spent in the Day Case Unit. The patient can stay as little as 20 minutes from their arrival to going home. This prospective pilot study was designed to evaluate the visual outcome, safety and patient satisfaction of "Cataract Surgery by Appointment".

## Methods

Patients are examined at the clinic, and when a visually significant cataract is diagnosed, the risks and benefits of cataract surgery are discussed with them. Then a choice is given to the patients either for "Cataract Surgery by Appointment" or conventional surgery. Having obtained an informed consent, biometry is performed and an appointment date and time given to the patient to present themselves to the Sussex Eye Hospital Theatre Suite for surgery. The patient is given a supply of gutt. cyclopentolate 1% and gutt. phenylephrine 2.5% eye drops. They are advised to instil them in the eye to be operated on, 2 hours prior to their surgical appointment, at half hourly intervals. Patients arrange their own transport. They are asked to have a completely clean face free of all cosmetics, and nails to be clear of varnish. When the patient arrives at the specified time and their identity has been confirmed, they are led to the operating theatre as soon as the previous case has been completed. Their identity is once again ascertained before their operation is performed by a Consultant Ophthalmic Surgeon (CSCL). Relatives or friends of the patient have the opportunity to view the procedure through a monitor at the waiting area, if all parties agree. The surgery is carried out under sub-Tenon's or topical anaesthesia. A theatre nurse instils gutt. Proxymetacaine in both eyes. The fellow eye also receives topical anaesthesia in case Povidone-Iodine disinfectant inadvertently enters it. Aqueous Povidone-Iodine 5% is instilled in the eye to be operated on before the drape is placed. The plastic material is divided and the speculum is used to open the eyelids and keep the lashes away from the eye. The scrubbed and masked surgeon then infuses sub-Tenon's anaesthesia unless the patient opted for topical anaesthesia alone. A scleral tunnel or corneal incision is performed. A curvilinear capsulorrhexis is performed, a 'divide and conquer' technique is usually used for phacoemulsification and the implant is usually an Acrysof^® ^MA60BM with a 6.0 mm optical diameter. The eye then receives a standard dose of sub-conjunctival cefuroxime. Patients are immediately discharged on a regimen of steroids and antibiotic eye drops four times daily and are reviewed the following day and a week later.

Patient selection was based on the ability to instil the drops by themselves, other members of family, or carers. They also had to arrange for their own transport. Patients unsuitable for local anaesthesia and those who preferred to undergo surgery under sedation were excluded from the study.

The patients were surveyed by a questionnaire to determine their satisfaction with the service and viability as a prospect for providing a more efficient cataract surgery service. The questionnaire groups the questions into those related to the clinic consultation, the time just before they come to hospital, their experience on arrival at the hospital, the operation itself, and their overall satisfaction (additional file 1). Patients were asked clearly to answer their questionnaire based on their experience from their first eye cataract surgery, with the questionnaires delivered to them after their operations.

## Results

Fifty-one eyes of 39 consecutive patients underwent "Cataract Surgery by Appointment" from 1^st ^February to 12^th ^December 2002. There were 16 male and 23 female. Forty-four (86%) patients received sub-Tenon's anaesthesia and 6 (14%) topical anaesthesia. A scleral tunnel was performed in 42 (82%) eyes and a clear temporal cornea incision in 9 (18%) eyes.

**Figure 1:**
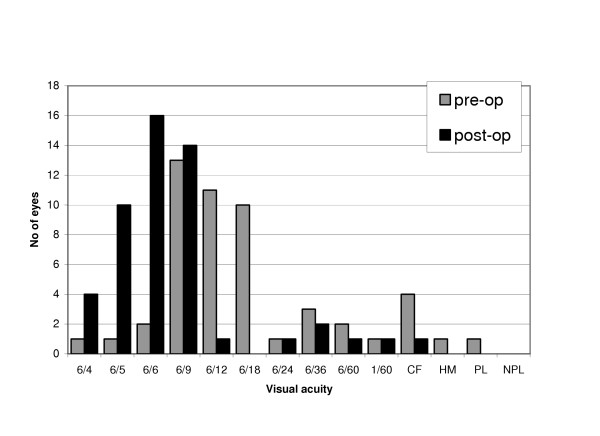
Comparison of preoperative versus postoperative best corrected visual acuity.

The pre-operative best-corrected visual acuity was 6/9 or better in 17 (33%) eyes with 4 (8%) eyes achieving 6/6 or better. The post-operative best-corrected visual acuity was 6/9 or better in 44 (86%) eyes with 30 (59%) eyes achieving 6/6 or better (Figure 1).

One eye had a best-corrected visual acuity that became worse post-operatively due to cystoid macular oedema; this patient's best-corrected visual acuity was reduced from 6/18 pre-operatively to 6/36 post-operatively.

Post-operative complications occurred in 3 (6%) eyes. Two eyes of the same patient developed Descemet's folds that settled with increased frequency of steroid eye drops, and there was one case of cystoid macular oedema (as already mentioned above). All patients went home directly from the operating theatre immediately following surgery. Patients were reviewed the day following surgery and at another follow up appointment 1–2 weeks post-operatively. The average number of days from surgery to discharge was 14.5 days.

Twenty-eight (72%) completed questionnaires were returned. Questionnaires were not sent again to the patients who did not reply, as we did not want them to feel pressurised. The results show that the majority of patients were satisfied with their overall experience of this mode of delivery for cataract surgery (Tables 1, 2, 3, 4).

**Table 1:** Level of satisfaction

	**Level of satisfaction (% of replies)**				
	**1**	**2**	**3**	**4**	**5**
	
**Explanation of surgery**	64	36	0	0	0
**Manner of staff**	75	25	0	0	0
**Facilities of waiting areas**	61	29	7	3	0
**Overall service**	72	14	14	0	0

Level of satisfaction

1 = very satisfied

2 = satisfied

3 = moderate satisfied

4 = dissatisfied

5 = very satisfied

**Table 2:** Level of discomfort

	**Level of discomfort (% of replies)**				
	1	2	3	4	5
	
**Discomfort during operation**	68	29	0	3	0

Level of discomfort

1 = no discomfort

2 = mild discomfort

3 = moderate discomfort

4 = painful

5 = very painful

**Table 3:** Other results

	**Yes (%)**	**No (%)**
**Adequate instructions for eye drops**	100	0
**Difficulty with drops**	14	86
**Self-administration of eye drops**	39	61
**Preference for nurse to instil eye drops**	18	82
**Preference for earlier arrival to theatres**	0	100
**Relatives/friends to watch surgery**	89	11
**Recommendation to a friend**	96	4

**Table 4:** Waiting time before surgery

	**No**	**<10 mins**	**<20 mins**	**<30 mins**	**>30 mins**
	
**Waiting time before surgery**	32%	30%	32%	3%	3%

(note this depends on whether patients arrive before their appointed time)

## Discussion

Well over 200,000 cataract operations are carried out per annum in the United Kingdom, making this one of the most common surgical procedures in the country [2]. In addition, small incision cataract extraction (phacoemulsification) is effective in terms of increase in vision and is safe with respect to the low incidence of surgical complications [3,4]. However, nationally the average patient wait was 8 to 10 weeks for an initial outpatient consultation and then a further seven months for surgery [1]. This called for long waiting times to be reduced and for services to be redesigned from the patients' point of view. As a result, one stop cataract surgery was introduced, but not without shortcomings [5], including difficulties in using theatre time efficiently, and without time for patients to consider whether to go ahead with surgery. We developed a new method of delivery of cataract surgery, "Cataract Surgery by Appointment", a modification of day case cataract surgery. We conducted the above pilot study as a prelude to a randomised controlled trial comparing "Cataract Surgery by Appointment" with Day Case Cataract Surgery.

Of note, no non-steroidal anti-inflammatory preparation such as diclofenac drops (Voltarol Ophtha) was used pre-operatively in this study. We felt it would be too complex to instil three different types of eye drops. In no case did the pupil come down in size during surgery. We are working on combining the dilating drops with diclofenac or similar in a single slow release preparation. In addition, there is now emerging evidence for the feasibility of per-operative intracameral administration of pupil dilating medication [6,7]. If this proves to be satisfactory, it would make "Cataract Surgery by Appointment" even more patient-friendly. Whilst it has proven to be the case that patients or their carers could be trusted to instil pre-operative dilating eyedrops, the availability of intracameral preparations for pupil dilation for per-operative use can also expand "Cataract Surgery by Appointment" to those patients who cannot instil eye drops themselves, for example due to rheumatoid arthritis.

In terms of patient safety the following apply. The fact that each patient arrives at an appointed time reduces the risk of operating on the wrong patient; there is only ever one patient on site at a time. Patients are fully awake without sedation, so we are in constant conversation with them as to what is about to happen. They know which eye is to be operated on and will have instilled dilating eye drops in that eye only. This reduces the risk of operating on the wrong eye.

"Cataract Surgery by Appointment" performed under local anaesthesia has obvious benefits for patients in reducing waiting time at the hospital on the day of operation. This frees up resources and is also popular with patients.

Routine medical testing before cataract surgery was not employed, as it does not measurably increase the safety of the surgery [8]. The per-operative and post-operative complications in our study compare favourably with the national rate of complications [9]. We had no case of posterior capsule rupture, vitreous loss or endophthalmitis.

Patient satisfaction is related to the patient's pre-operative expectations and the quality of care given during the hospital stay and follow-up [10]. Satisfaction with the process of treatment and facilities was high. Most patients were satisfied with every aspect on which they were questioned. Patient satisfaction may be further improved if Day 1 review could be omitted. We have already started to omit Day 1 review for second eye surgery, but as the time spent in hospital on the day of their operation is so short, it is necessary to spend some time to explain how to use their post-operative eye drops, what activities to avoid, and what to do if symptoms suggestive of a serious post-operative complication should occur. We feel this may be better done on Day 1, but it is possible that the same effect could be achieved by Day 1 telephone review.

"Cataract Surgery by Appointment" performed under local anaesthesia gave a high level of patient satisfaction. This method of delivery of cataract surgery is safe, effective, and is the ultimate form of day case cataract surgery for highly selected cases, when performed by a skilled ophthalmic surgeon. We recommend this method of delivery of cataract surgery to surgeons who are competent, consistent in speed, and have a low rate of complications. Attention should be paid to risk-stratification, so complex cases are allocated more time on the operating list [11,12]. This type of delivery of cataract surgery is probably not suitable for teaching lists. It may be particularly suited to hospitals where the ward or day case unit are physically remote from the operating theatre.

## Competing interests

The author(s) declare that they have no competing interests.

## Pre-publication history

The pre-publication history for this paper can be accessed here:


